# Evaluation of Plaque Characteristics and Inflammation Using Magnetic Resonance Imaging

**DOI:** 10.3390/biomedicines9020185

**Published:** 2021-02-12

**Authors:** Kristina Andelovic, Patrick Winter, Peter Michael Jakob, Wolfgang Rudolf Bauer, Volker Herold, Alma Zernecke

**Affiliations:** 1 Institute of Experimental Biomedicine, University Hospital Würzburg, 97080 Würzburg, Germany; 2 Experimental Physics V, University of Würzburg, 97074 Würzburg, Germany; patrick.winter@physik.uni-wuerzburg.de (P.W.); peja@physik.uni-wuerzburg.de (P.M.J.); vrherold@physik.uni-wuerzburg.de (V.H.); 3 Internal Medicine I, Cardiology, University Hospital Würzburg, 97080 Würzburg, Germany; Bauer_W@ukw.de

**Keywords:** atherosclerosis, mouse models, wall shear stress, pulse wave velocity, arterial elasticity, inflammation, magnetic resonance imaging

## Abstract

Atherosclerosis is an inflammatory disease of large and medium-sized arteries, characterized by the growth of atherosclerotic lesions (plaques). These plaques often develop at inner curvatures of arteries, branchpoints, and bifurcations, where the endothelial wall shear stress is low and oscillatory. In conjunction with other processes such as lipid deposition, biomechanical factors lead to local vascular inflammation and plaque growth. There is also evidence that low and oscillatory shear stress contribute to arterial remodeling, entailing a loss in arterial elasticity and, therefore, an increased pulse-wave velocity. Although altered shear stress profiles, elasticity and inflammation are closely intertwined and critical for plaque growth, preclinical and clinical investigations for atherosclerosis mostly focus on the investigation of one of these parameters only due to the experimental limitations. However, cardiovascular magnetic resonance imaging (MRI) has been demonstrated to be a potent tool which can be used to provide insights into a large range of biological parameters in one experimental session. It enables the evaluation of the dynamic process of atherosclerotic lesion formation without the need for harmful radiation. Flow-sensitive MRI provides the assessment of hemodynamic parameters such as wall shear stress and pulse wave velocity which may replace invasive and radiation-based techniques for imaging of the vascular function and the characterization of early plaque development. In combination with inflammation imaging, the analyses and correlations of these parameters could not only significantly advance basic preclinical investigations of atherosclerotic lesion formation and progression, but also the diagnostic clinical evaluation for early identification of high-risk plaques, which are prone to rupture. In this review, we summarize the key applications of magnetic resonance imaging for the evaluation of plaque characteristics through flow sensitive and morphological measurements. The simultaneous measurements of functional and structural parameters will further preclinical research on atherosclerosis and has the potential to fundamentally improve the detection of inflammation and vulnerable plaques in patients.

## 1. Introduction

Atherosclerosis is a chronic, inflammatory disease of the vessel wall, characterized by intimal lesions (atherosclerotic plaques) in the vascular system. Rupture or erosion of these plaques can trigger disease such as myocardial infarction and stroke, the major causes of morbidity and mortality worldwide. Plaque development is driven by the intimal accumulation of leukocytes. Early endothelial dysfunction and lipid deposition trigger the recruitment of inflammatory cells and foam cell formation within intimal atherosclerotic plaques. In addition to monocytes/macrophages, also other immune cells, including T and B cells, contribute to the cellular plaque infiltrate. Increasing evidence supports a critical role of inflammation and the immune system in atherosclerosis in preclinical mouse models of atherosclerosis and in patients, as reviewed in detail in [1,2,3,4]. Clinical trials evaluating the therapeutic targeting of the inflammatory cytokine interleukin-1β (CANTOS trial) or low-dose therapy with anti-inflammatory Colchicine in patients with chronic coronary disease demonstrated a reduction in the risk of developing adverse cardiovascular events [5,6]. However, the tools to detect inflammation and processes in the vessel wall that could indicate disease progression, or the immanent risk of plaque erosion or rupture are still limited, and subject of continuous research and improvements based on e.g., the technical advancement of imaging tools, which help to increase the diagnostic quality and minimize the burden caused by diagnostic procedures on patients and laboratory animals.

Imaging of inflammation for clinical and preclinical applications has been achieved using various probes for positron emission tomography (PET) imaging or combined PET/computed tomography (PET/CT) scanning. In a PET study using ^18^F-fluorocholine (^18^F-FCH), a strong correlation was found between the uptake of the tracer and the macrophage infiltration, as revealed by histological analysis of carotid endarterectomy specimens [7]. Other examples of PET/CT include ^68^Ga-DOTATATE PET/CT to image somatostatin receptor expression, initially developed for imaging of tumors, as a tool to detect inflammation of large arteries [8], 18F-4V for imaging of VCAM-1 (vascular cell adhesion molecule-1) expression in atherosclerosis [9], or CD80-targeting PET Tracers [10]. These techniques offer non-invasive detection of inflammation at high spatial resolution but impose a high radiation burden. In addition, PET in combination with magnetic resonance imaging (PET/MRI) has been tested in animal models of atherosclerosis and in humans, as reviewed in [11]. For instance, P-selectin was targeted by gallium-68-labeled fucoidan PET for non-invasive characterization of vulnerable plaques in apolipoprotein E-deficient (ApoE^−/−^) mice [12], and an integrated ^18^F-fluorodeoxyglucose-PET (FDG-PET) and dynamic contrast enhancement MRI (DCE-MRI) imaging protocol was applied to noninvasively quantify lesion inflammation, neovasculature, permeability, and plaque burden in a rabbit model of atherosclerosis [13]. Moreover, large-scale multiterritorial PET/MRI demonstrated ^18^F-FDG uptake in plaque-free arterial segments as an indicator of early stages of atherosclerosis [14]. PET/MRI offers a good soft tissue contrast; however, patients are still exposed to radiation due to the PET tracer. Therefore, the development of completely non-invasive imaging techniques is still of high relevance in order to reduce the examination burden on the laboratory animals and to increase patient comfort. Flow-sensitive MRI is an excellent imaging modality as it provides the assessment of functional cardiovascular parameters such as pulse wave velocity and wall shear stress (WSS) without the use of ionizing radiation. Due to its non-invasiveness, it is also well suited to long-term research studies to examine the morphological and functional changes involved during atherosclerotic plaque development.

Here we explore the applications and potential of MRI flow quantification to evaluate atherosclerotic lesion formation and progression. In particular, parameters such as wall shear stress and pulse wave velocity assessed by flow-sensitive MRI may serve as surrogate markers for the characterization of plaques. In addition, MRI provides an excellent tissue contrast and, therefore, enables combined measurements of flow related parameters as well as imaging of the vessel wall, plaque morphology and inflammation. As it provides 4D spatial and velocity measurements it is, therefore, particularly suitable for measuring complex arterial geometries such as the aortic arch. Therefore, MRI is a potential tool, which may help to answer several questions of preclinical research. In particular, a combination of flow-sensitive measurements with the assessment of plaque morphology and inflammation could lead to a better characterization of atherosclerotic plaques, an early detection of vessel wall inflammation, and help to identify plaque vulnerability. 

## 2. The Role of Shear Stress in the Development and Progression of Atherosclerosis

Vascular inflammation is intricately linked to the biophysical forces acting on the vessel wall through the blood flow, which exerts a tangential force vector, causing a shearing deformation of the vessel wall. The shear stress generated, i.e., the fluid frictional force acting at the apical surface of endothelial cells, can be separated in a longitudinal part along the main direction of the flow and a circumferential component that can be attributed to helical flow [15]. At predilection sites in particular like the inner curvature of arteries, bifurcations and branchpoints, the resulting shear stress is low and oscillatory, favoring inflammation and remodeling processes and, therefore, plaque development and growth [16,17]. The growing plaque further disturbs flow patterns, leading to regions exposed to even lower and higher WSS as well as oscillations in the direction of flow, which can be characterized via the oscillatory shear index (OSI). Numerous effects of shear stress are described, as reviewed in detail in [18,19,20]. Shear stress is sensed by luminal endothelial mechanoreceptors, which trigger a complex network of intracellular pathways to control endothelial cell homeostasis. In particular, low endothelial shear stress disrupts nitric oxide-dependent vascular protection, triggering endothelial cell activation, low-density lipoprotein (LDL) cholesterol uptake, and endothelial permeability. Activated endothelial cells upregulate adhesion molecules and cytokines to drive leukocyte recruitment to the vessel wall [18,19,20,21]. The production of reactive oxygen species is augmented by low endothelial shear stress, which can cause LDL particles deposited in the vascular wall to undergo oxidative modification to further potentiate their pro-inflammatory impact on endothelial cells and other vascular and immune cells accumulating in the artery wall. Low endothelial shear stress is also known to sensitize endothelial cells towards interleukin-1β [22], a process that could further amplify local inflammatory processes. Monocytes recruited to the vessel wall differentiate into macrophages, which ingest lipids and transform into foam cells. In the context of ongoing inflammation, vascular smooth muscle cell (SMC) mitogens are produced, promoting SMC dedifferentiation, and SMC migration from the media into the intima and their proliferation, a key process that controls the formation of a fibrous cap [18]. Low wall shear stress has been correlated to a reduction in SMC and collagen production, and an increased matrix metalloproteinase (MMP) activity [22], contributing to a degradation of the extracellular matrix [18]. 

Mouse models of atherosclerosis confirmed the determinant role of low shear stress for atherosclerotic lesion size and vulnerability, whereas effects of oscillatory shear stress may be more complex and conflicting results have been reported. A perivascular shear stress modifier placed around murine carotid arteries to investigate effects of fluid shear stress on plaque formation and composition revealed that low shear stress induced larger lesions with a vulnerable plaque phenotype, whereas vortices causing oscillatory shear stress induced stable lesions [23]. Lesions in lowered shear stress regions furthermore showed more outward vascular remodeling, an increased lipid content and matrix metalloproteinase activity compared to oscillatory shear stress lesions [23], which seems to be regulated by an increased expression of multiple chemokines [24]. In another study using cuff-induced atherosclerosis, computational fluid dynamics suggested that shear stress magnitude contributes to the formation of advanced plaques with a vulnerable phenotype, whereas variations in both magnitude and direction promoted the formation of plaques with stable features [25]. In a study investigating the temporal and spatial changes in WSS over a growing plaque and a potential correlation of WSS and plaque composition, lumen narrowing was observed in all mice after cuff placement. As the plaque developed and intruded into the lumen, absolute WSS significantly decreased and the proximal part of the plaque exposed to relatively lower WSS was small and eccentric, and harbored a greater accumulation of macrophages in histological sections [26]. Interestingly, low shear seemed to induce pro-inflammatory M1 macrophage polarization in murine thin-cap atherosclerotic plaques induced by cast placement. In macrophage-rich areas of low shear stress-induced lesions, inflammatory M1 markers were highly expressed, while pro-healing M2 markers were elevated in lesions exposed to oscillatory shear stress [27]. Another study conducted in apolipoprotein E-deficient (ApoE^−/−^) mice using cuff placement found a significantly higher MMP-2 and MMP-9 activity in the upstream region of the cuff exposed low shear stress, whereas a more stable plaque phenotype was reported in the region of oscillatory shear stress [28], pointing out the connection of wall shear stress and vascular remodeling processes through MMP activity. 

Transglutaminase activity may contribute to regulate plaque composition by controlling monocyte recruitment, as investigated in another cuff model of atherosclerosis. ApoE^−/−^ mice treated with a transglutaminase inhibitor showed a significant reduction in lipid and macrophage content in the distal region of the cast, where the shear stress is oscillatory. Interestingly, in these regions the lesion size was increased due to an augmented smooth muscle cell content [29]. In in vitro experiments of bovine SMCs exposed to oscillatory shear stress, an increased vascular smooth muscle cell proliferation and activation of the PI3K-Akt signal transduction pathway was observed [30], whereas laminar shear inhibited SMC proliferation [31]. In endothelial cells, however, oscillatory shear leads to endothelial dysfunction and altered lipid uptake as investigated by different in vitro and in vivo studies [32,33,34,35], pointing out the ambiguous role of oscillatory shear stress in the context of atherosclerosis. 

Interestingly, wall shear stress is influenced by systemic blood pressure. Computational fluid dynamics demonstrated that ivabradine-induced heart rate reduction enhanced the WSS in the aorta, accompanied by an induction of eNOS (endothelial NO synthase) and a suppression of VCAM-1 at the inner curvature of the aorta [36]. In a rabbit model of atherosclerosis, it was reported that the heart rate reduction with ivabradine was furthermore associated with a more stable plaque phenotype with decreased macrophage content, plaque microvasculature flow and permeability [37]. Moreover, it was shown that lowered blood pressure and improved left ventricular function induced by docosahexaenoic acid supplementation reduced oscillatory shear at ostia in the descending aorta, which led to decreased plaque endothelial IL-1β expression and a reduction in lesion burden in distal aortas and in brachiocephalic arteries of atherosclerosis-prone mice [38].

### 2.1. Plaque Characteristics, Cardiovascular Risk and the Role of Wall Shear Stress (WSS)

Plaque characteristics have also been evaluated in different studies in patients. MRI of carotid artery plaque burden and characteristics were analyzed in a cohort of 1256 participants from the Atherosclerosis Risk in Communities (ARIC) carotid magnetic resonance imaging sub study. In this study, the presence of a lipid core was independently associated with incident cardiovascular disease events when adjusted for traditional cardiovascular disease risk factors and carotid artery wall thickness in asymptomatic individuals, and improved risk prediction of incident cardiovascular disease events over traditional cardiovascular risk factors [39]. Of note, the majority of acute coronary syndrome culprit lesions displayed a ruptured fibrous cap, and only 25% displayed an intact fibrous cap, as revealed by optical coherence tomography. Culprit lesions with intact fibrous caps displayed a lower lipid content, less calcification, a thicker fibrous cap and were largely localized near coronary bifurcations. Moreover, they also showed an enrichment in CD4^+^ and CD8^+^ T cell, providing insights into a pathogenesis mechanism involving cells of the adaptive immune system and their effector molecules [40]. 

How wall shear stress affects advanced atherosclerosis and plaque vulnerability in patients, however, is still unclear and both high and low wall shear stress have been suggested to play a detrimental role and to associate with aspects of plaque progression and vulnerability in clinical imaging studies. In different studies, for instance, it was reported that plaques rupture predominantly in the upstream region of the plaque shoulder [41,42], where plaques are exposed to an increased WSS, suggesting that an elevated WSS promotes plaque vulnerability in coronary atherosclerosis [43]. In line, increased shear stress was reported to cause vulnerable plaque formation by inducing angiogenesis [44]. Furthermore, focal elevations of high wall shear stress have been associated with plaque rupture and future myocardial infarctions [22,45]. In a three-dimensional intravascular ultrasound study, Fukumoto et al. also found a relation of localized elevations in the shear stress and coronary plaque rupture. In this study, however, absolute WSS values were demonstrated to not directly provoke mechanical destruction of the fibrous cap, but to play a triggering role in fibrous cap rupture [45]. A recent study from Kojima et al. has also shown the association of high maximum wall shear stress and plaque rupture in aortic atherosclerosis [46]. High wall shear stress values were furthermore associated with high-risk atherosclerotic plaque characteristics in coronary artery disease patients independent of stenosis severity [47]. A high endothelial shear stress gradient was moreover associated with plaque rupture and erosion, whereas a high oscillatory shear index correlated with plaque erosion, as revealed by intracoronary optical coherence tomography [48]. 

However, both in humans [49,50,51,52,53,54] and porcine models of atherosclerosis [55,56], plaque vulnerability was also linked to low shear stress. Low wall shear stress may predict lesion progression to require percutaneous coronary intervention and to be associated with future angiographically driven revascularization and non-culprit major adverse cardiac events [49]. In an intracoronary 3D optical coherence tomography study, Chatzizisis et al. found local, low WSS and expansive lumen remodeling to be associated with the presence of high-risk plaques. Furthermore, they introduced a shear stress score, a global metric of the portion of the artery exposed to the lowest wall shear stress rates. An increased shear stress score was linked to an increased frequency of high-risk plaques [50]. Moreover, a larger lipid burden, a thinner fibrous cap and a higher prevalence of thin cap fibroatheroma in regions exposed to low shear stress was observed in a combined optical coherence tomography and computational fluid dynamics study [51]. Low WSS, furthermore, predicted the localization of high-risk plaques in an intravascular ultrasound and histopathology study in diabetic, hypercholesterolemic pigs. The complexity and heterogeneity of these lesions was determined by the magnitude of low shear stress [55], which seems to favor the focal evolution of thin-capped atheroma by promoting an imbalance of decreased collagen synthesis and increased collagen breakdown [56]. Also in humans, atherosclerotic plaque burden, composition, and distribution was linked to low wall shear stress in patients with coronary artery disease, as revealed by virtual histology intravascular ultrasound (IVUS) and Doppler velocity measurements for computational fluid dynamics modeling [52]. Moreover, local low shear stress was shown to have a critical effect on de novo eccentric plaque development and progression [53]. In another study, it was observed that artery segments exposed to low WSS showed total plaque progression. In contrast, exposure to low and oscillatory WSS caused total plaque area regression but was associated with a phenotypic transformation towards a more vulnerable phenotype due to a decrease in fibrous tissue and an increase in necrotic core and calcium deposition. This may indicate that low in combination with oscillatory wall shear stress to be the dominant flow characteristic affecting plaque progression and vulnerability [54].

These in part contradictory reports of the role of WSS in plaque development, progression and rupture emphasize the need for further studies to resolve causality. Most of the studies aiming at a local plaque characterization, however, rely on invasive intra-artery measurements, such as intracoronary optical coherence tomography or intravascular ultrasound, which can cause severe complications, including ventricular fibrillation or vessel dissection, or harbor limitations such as the investigation of only a localized area of the artery. The exploration of alternative and non-invasive methods is such warranted. Flow-sensitive MRI provides the non-invasive assessment of time-resolved 3-dimensional velocity fields and high-resolution morphological information simultaneously in all places of the vascular tree. In particular, a combination of time-resolved 3D spatial and velocity encoding (4D-phase contrast) MRI is considered to drastically improve non-invasive diagnostics, since it allows flow measurements at arbitrary locations and does not depend on the positioning of predefined 2D slices, which may help to reduce the inter-observer variability. MRI could thus be a potential tool for in vivo measurements of wall shear stress and the oscillatory shear index, and by allowing a 4-dimensional view of the cardiovascular hemodynamics help to better understand and characterize the pathology of atherosclerosis. It may also present a possible surrogate for inflammation imaging since no contrast agents or tracers are required.

### 2.2. Magnetic Resonance Imaging (MRI) Measurements of Wall Shear Stress (WSS) and the Oscillatory Shear Index (OSI)

Moore et al. conducted one of the first MRI-based in vivo wall shear stress measurements in humans in 1994 [57]. Since then, 4D MR techniques for WSS quantification have become increasingly important for clinical applications in the aorta [58,59,60,61]. In 2006, 2D phase contrast MRI (PC-MRI) and computational fluid dynamics (CFD) using the Navier–Stokes equation were used for the first time for investigating the WSS in mice and other mammals [62]. In this work, an allometric scaling law for the WSS of the form WSS ~ M^b^ (M: body mass, b = −0.38) was found empirically. Weinberg et al. subsequently stated that the WSS inversely depends on body mass to the 3/8th power and predicted 20-fold higher WSS values in mice relative to men [63]. However, early computational models did not take pulsatile flow into account. Therefore, recent models considering more physiological oscillating flow predicted a men-to-mouse-ratio of only 1:8 [64] and, therefore, lower WSS values. CFD-aided computations of murine WSS were also used by Feintuch et al. [65]. Here, 2D MRI flow measurements were compared with ultrasound measurements. It was observed that ultrasound flow quantification overestimates flow and stated that the MRI flow measurements were slightly more accurate. Furthermore, the CFD simulations revealed that the asymmetry of the WSS distribution across the arch is dependent on the Reynolds number of blood. Subsequently, MRI and CFD were utilized by Doormaal et al. [66] using 2D flow measurements in the aortic root as input function for the CFD simulation. Furthermore, a combination of 4D flow MRI and CFD was recently introduced for detailed WSS studies in the aortic arch of rabbits [67]. 

While CFD models used for WSS calculations provide valuable information about the complex hemodynamics in the mouse, they still exhibit some limitations. For instance, CT scans of 3D casts of the aorta, which required sacrificing of the animal [65,66] were used to assess the geometry information for the simulations. Thus, CFD models usually neglect lumen area changes and aortic compliance and, therefore, can overestimate the WSS [68,69]. An alternative approach to assess WSS are direct measurements using flow MRI. These techniques provide direct flow and geometry information from the magnitude and phase data. However, as demonstrated in a study in humans [15] and in a flow phantom [70], direct WSS measurements are highly dependent on the spatial and temporal resolution, making the development of preclinical techniques challenging.

First, 2D techniques using triggered radial PC MRI [68] or Cartesian flow cine MRI [71] were developed to study WSS in the abdominal aorta of wild-type, ApoE^−/−^- and Low-density lipoprotein receptor-deficient (Ldlr^−/−^) mice. WSS was also assessed in the carotids of mice with unstable plaques using Gd-enhanced 2D flow MRI [72] and in the carotids of rats [73]. Furthermore, 2D flow MRI was used to determine WSS at different locations along the vascular tree in arteries and veins in wild-type mice, revealing significant sex- and age-related differences of the WSS and flow values [74]. A first 4D PC MRI technique for direct WSS measurements in the mouse was presented in 2011 [75]. Janizcek et al. used 3D spiral MRI with an isotropic spatial resolution of 170 µm and a scan time of 60 min to study the longitudinal and circumferential WSS as well as the oscillatory shear index (OSI) in the aortic arch of 24-week-old ApoE^−/−^ mice. A highly asymmetric distribution of longitudinal WSS was observed, yielding the lowest values near the inner radius of the arch, where atherosclerotic plaques predominantly manifest. An opposed relation was observed for the circumferential WSS, yielding the highest values near the inner radius. For preclinical WSS measurements in mini pigs, a retrospectively triggered radial 4D PC MRI technique was developed by Wentland et al. [76]. This method enabled simultaneous measurements of WSS and pulse wave velocity (PWV) in the abdominal aorta. In 2017, Braig et al. presented a triggered Cartesian 4D PC MRI technique using a cryogenic surface coil for WSS measurements in the murine aortic arch [77]. In this work, dummy scans were used to maintain a close to steady state condition of the longitudinal relaxation in order to reduce image artifacts. For the first time, streamline presentations of aortic flow in the murine aorta were presented. The presented 4D flow technique featured scan times of 40 min and a moderate spatial resolution (300 × 320 × 280 µm^3^). Therefore, to accelerate the measurement and increase spatial resolution, a retrospectively triggered radial 4D PC MRI technique and compressed sense image reconstruction were introduced to further improve the measurement [78]. 

**Figure 1:**
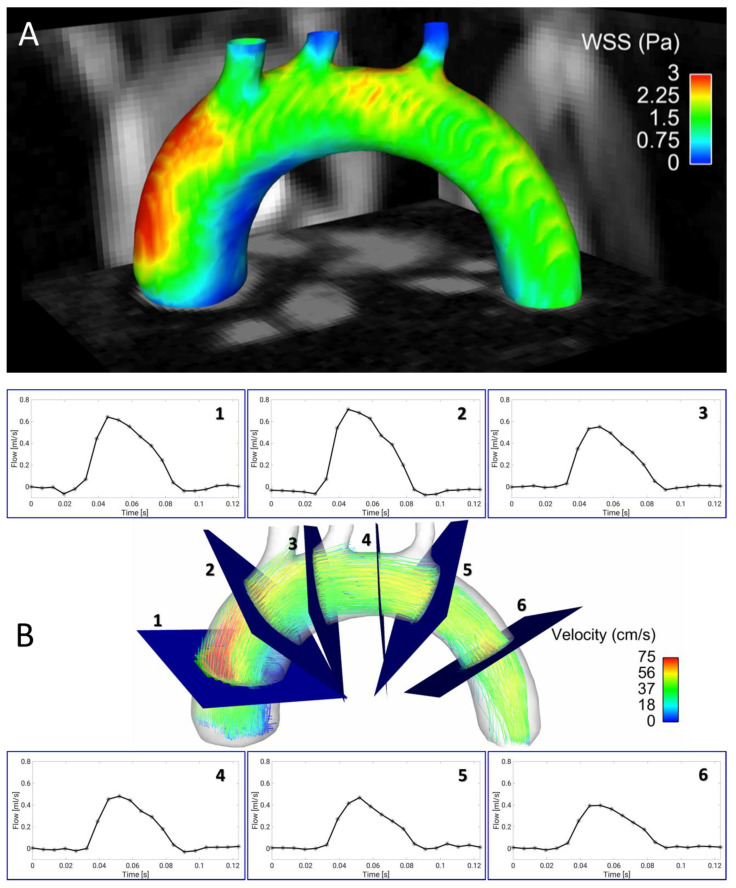
Four-dimensional (4D) flow and wall shear stress (WSS) measurements in the murine aortic arch. (**A**) Wall shear stress map of the aortic arch of an ApoE^−/−^ mouse fed a Western diet, assessed with a self-gated radial 4D phase-contrast magnetic resonance imaging (PC-MRI) sequence [79]. Especially in the ascending aortic arch, a large region of low WSS can be observed in the inner curvature. This area is known to be prone to plaque development. (**B**) Streamline presentation of the measured flow and through plane flow for 6 exemplary analysis planes, obtained from the same 4D flow measurement [79,80].

Most imaging techniques for wall shear stress quantification rely on an electrocardiogram (ECG)-based synchronization of the measurement with the animal’s cardiac motion, which can be prone to interferences with the imaging gradients. Furthermore, 4D flow measurements are still very time-consuming. Therefore, accelerated techniques are needed in order to assess arterial wall shear stress values in reasonable measurement times. In 2019, a radial PC MRI sequence was presented that uses self-navigation and retrospective cine reconstruction for 4D flow measurements in the murine aortic arch [79]. Due to the strong signal enhancement of blood and suppression of undersampling artifacts, a scan time reduction to 35 min, and high spatial resolution (100 µm isotropic) were achievable. The new technique was used to study the 3D distribution of WSS values across the aortic arch (see Figure 1A), temporal changes of WSS over the cardiac cycle and the oscillatory shear index (OSI). However, in addition to shear stress, other factors such as local wall architecture and compliance will have to be considered to fully characterize atherosclerotic lesions in atherosclerotic vessels [69].

## 3. Arterial Wall Stiffness in Atherosclerosis

During atherosclerosis progression, the vessel wall shows structural and morphological adaptions to vascular stress and inflammation. Compensating for a higher circumferential stress due to hypertension, an augmentation in collagen content and number of SMCs is observed, leading to an increased wall thickness [81]. In particular, circumferential stress was shown to stimulate the NADPH-oxidase Isoform 1 (Nox1)-dependent formation of reactive oxygen species (ROS) [82], resulting in a dysfunction of SMCs and the dedifferentiation from the contractile to the synthetic and migratory phenotype as well as in an increase in MMP activity [83]. Chesler et al. have also shown an increased production and activation of matrix-degrading MMP-2 and MMP-9 in ex vivo porcine carotid arteries as a consequence of elevated transmural pressure [84]. Hypertension and a decreased aortic compliance due to reduced elastin content, however, does not seem to affect atherosclerotic plaque burden in atherosclerosis-prone mice [85], suggesting that reductions in the aortic wall compliance may not directly promote atherosclerosis, but rather function as indicators of the disease. However, it has also been shown that estrogen-dependent inhibition of arterial stiffening provided protection against atherosclerosis in females. In this study, oxidized LDL stimulated the secretion of MMP12 in macrophages, which was antagonized by estrogen. Reduced MMP-12 expression led to reduced aortic stiffness and inhibited atherosclerosis [86].

Several human studies investigated the correlation between atherosclerosis and the stiffness of the aortic arch and the carotid artery, and therefore the importance of PWV for early detection of cardiovascular disease. In 2000, the Rotterdam study showed that a high intima media thickness causes an increased arterial stiffness, which in turn lead to an increased plaque burden. They further found a connection between peripheral artery disease and an increased stiffness of the aorta and carotid arteries [87]. Interestingly, in MESA (Multi-Ethnic Study of Atherosclerosis) including 3527 participants, aortic arch PWV measured by MRI at baseline and when patients were free of overt cardiovascular disease predicted cardiovascular disease events among middle-aged (45–54 years old) individuals during a 10-year follow-up period, whereas the aortic arch PWV was not associated with cardiovascular disease among elderly [88]. At early stages of atherosclerosis, when lumen narrowing is still absent or minimal, the measurement of the PWV may thus function as a surrogate marker for early atherosclerosis. This notion was also supported in a study in atherosclerosis-prone mice, revealing an increased PWV before morphological vessel wall changes and wall-thickening occurs [89]. But measuring the PWV may also be useful in characterizing atherosclerotic plaques. For example, Harbaoui et al., developed a method for measuring the coronary PWV (coPWV) to investigate the effect of local coronary stiffness and found a significant difference in coPWV values in stable and unstable coronary vessel diseases. Furthermore, the coPWV showed an association with acute coronary events in contrast to the aortic PWV (aoPWV) [90]. The local and cell-specific biological responses of the arterial wall and how these are linked to plaque inflammation and vulnerability, however, are not fully understood in detail. 

### 3.1. Measurements of Pulse Wave Velocity (PWV)

Local elasticity (Et) is related to the pulse wave velocity c (PWV) through the Moen–Korteweg equation [91]:

c = Eth/(ρD),(1)

where h is the vessel thickness, D the vessel diameter and r the blood density. During atherosclerosis progression, the vessel wall thickness (h) increases and the diameter decreases (D), consequently leading to an increase in PWV (c). Therefore, the PWV represents an important physical parameter to characterize the mechanical properties of arteries. The gold standard and most accurate method for measuring the pulse wave velocity are invasive methods with catheter-based flow or pressure probes. These have the highest accuracy and can, therefore, show new insights into cardiovascular diseases. However, as the use of catheters imply a surgical procedure, the invasive PWV assessment is complicated and laborious due to the intravascular placement of probes, limiting the use in human and animal studies. Besides the use of invasive pressure probes, applanation tonometry [92] and Doppler ultrasound (US) are the most common and established methods for PWV estimation in clinics. In US-based PWV acquisitions, ultrasonic waves are used to measure flow. Two probes with a known distance are placed on the target arteries, where one ultrasonic wave is transmitted in direction of the flow and one against the flow. The difference in transit time is directly proportional to velocity and by dividing the measured distance by the difference in transit time of the two waveforms, the PWV can be calculated. An alternative ultrasound-based method is pulse wave velocity imaging (PWI), which uses the detection of vessel wall motion [93]. In the clinic, however, the evaluation of the carotid-femoral PWV (cfPWV) and the brachial-ankle PWV (baPWV) are most common [94]. Ultrasound is, furthermore, used to characterize plaques in carotid artery disease patients in vivo [95] and to determine the local PWV in the human ascending aorta [96]. Also, for preclinical research of cardiovascular disease in animal models, new methods are constantly being developed. For example, Wang et al. developed a new methodology for PWV quantifications in the rat abdominal aorta, which was further validated using invasive measurements [97]. Furthermore, micro-ultrasound measurements revealed alterations of the PWV in ApoE^−/−^ mice fed a high-fat diet [98]. 

Ultrasound-based PWV measurements offer many advantages: they are non-invasive, inexpensive and do not require radiation. Moreover, as it provides flow measurements with a very high temporal resolution, it enables real-time measurements of pressure and flow waves in the vasculature. Not only the PWV but also the WSS [99,100] and the vessel wall thickness [101] can be assessed with ultrasound-based methods, enabling a good characterization of the flow and the vessel morphology in one session. However, the estimation of the measured vessel distance is not always correct. A small error in the distance measurement can already lead to high inaccuracies in PWV estimation with up to 30% error [102]. Furthermore, cfPWV and baPWV only provide global information about the vessel properties. Many studies have already indicated that local PWV measurements are needed to provide a more precise evaluation of the artery conditions [103,104]. Assessing the local stiffness in a vessel segment may be important to identify plaque formation, but also to characterize the hemodynamic environment of progressive (vulnerable) plaques. The local determination of PWV with ultrasound in various target arteries was already described [94,96,105,106]. However, its wide use in the clinic is still limited. Di Lascio et al. described an ultrasound-based method for the determination of the local PWV in the murine abdominal aorta and found significant differences between younger and older wild-type mice [107]. Moreover, a good correlation between regional transit-time (TT) and local flow-area (QA) method of ultrasound-based PWV assessment was found [108], proving the feasibility of determining the global and local PWV in the murine system with ultrasound. 

Despite all the advantages, ultrasound-based PWV measurements also entail some detrimental characteristics like the error-prone distance measurements [109]. Therefore—especially in comparison to MRI—ultrasound is very user-dependent [110]. In addition, ultrasound has a limited penetration depth, making some vessels difficult to reach. Furthermore, ultrasound only provides a comparably poor soft tissue contrast in comparison to MRI, which not only provides flow information but also high-resolution morphological images. Ultrasound flow measurements also tend to overestimate the peak flow values [65]. For that reason, the development of MRI based PWV measurements is of great interest.

### 3.2. MRI-Based PWV Measurements

The quantification of the PWV with MRI was firstly described in 1989 by Mohiaddin [111]. Today, its determination using phase contrast cine MRI and transit time methods is already established for clinical in vivo measurements. In contrast to MRI based elastography measurements [112], PWV quantification provides aortic stiffness values without the need for additional hardware. PWV is regarded as a potential predictor of cardiovascular events, which is used for the assessment of aortic stiffness in aortic stenosis [113] and of the carotid arteries [114]. Recent technical advancements also enable real-time PWV measurements, which can be applied during exercise stress testing [115]. In preclinical atherosclerosis research, mouse models are of great relevance to reveal the pathophysiological mechanisms underlying atherogenesis and plaque progression, and to characterize the therapeutic effects of new drugs. The development of atherosclerotic plaques can be accelerated by feeding a cholesterol-rich and high-fat diet, enabling the investigation of atherogenesis in a reasonable period. Therefore, there is a need for developing methods for the indirect measurement of vascular elasticity and function in mice. The adaption of these methods to the murine system is challenging due to the small vessel dimensions, the high heart rates (cardiac periods ~120 ms) and the small aortic arch dimensions (around 2 mm diameter and 30 mm length), requiring both, high temporal and spatial resolution methods. Consequently, the needed scan time is markedly increased in order to compensate for the loss of signal-to-noise ratio (SNR) in comparison to standard PC-MRI sequences. Zhao et al., who presented a method based on radial phase-contrast MRI to determine the global PWV and WSS in the murine abdominal aorta [68], described the first in vivo implementation of PWV measurements with MRI in atherosclerotic mice. Simultaneously, Parczyk et al., have developed a high-field MR-method for assessing the global PWV in a vessel segment based on travel-time-measurements of the pulse wave [116]. In a swine model with familial hypercholesterolemia, the global PWV was assessed with an undersampled radial 4D-PC cine sequence with retrospective ECG gating. Here, it was shown that the obtained values correlated well with invasive pressure probes measurements. Moreover, it was demonstrated that one single 4D-PC MRI measurement was sufficient to determine both PWV and WSS in only 10 min [76].

The evaluation of the vessel wall, e.g., for the identification of initial atherosclerosis, however, requires a technique to determine the local vascular elasticity as an early biomarker. Through the interrelation between vascular wall elasticity and intravascular hydrodynamic, the local PWV can be calculated through the local temporal behavior of the vessel cross section A and the corresponding volume flow Q with the equation:

c = ΔQ/ΔA.(2)

This MRI-based method of assessing the local PWV was shown for the first time in humans by Vuillémoz et al. [117]. As the mouse represents a good model for longitudinal studies of arterial elasticity and plaque development in atherosclerosis research, the determination of the local PWV was further adapted for the first time to the murine system [118]. With an accuracy of 5–10%, changes in vascular elasticity were observed in ApoE^−/−^ mice in comparison to wild type mice. Moreover, a significant increase in the PWV in the ascending aorta was shown even before a visible thickening of the vessel wall. Interestingly, this early loss of elasticity also correlated with an increased elastin fragmentation [89]. A direct comparison of local and global PWV values, however, revealed no significant correlation between global and local PWV values in ApoE^−/−^ mice, pointing out the heterogeneous distribution of arterial stiffening in early atherosclerosis and proving the importance of local PWV measurements [119].

**Figure 2:**
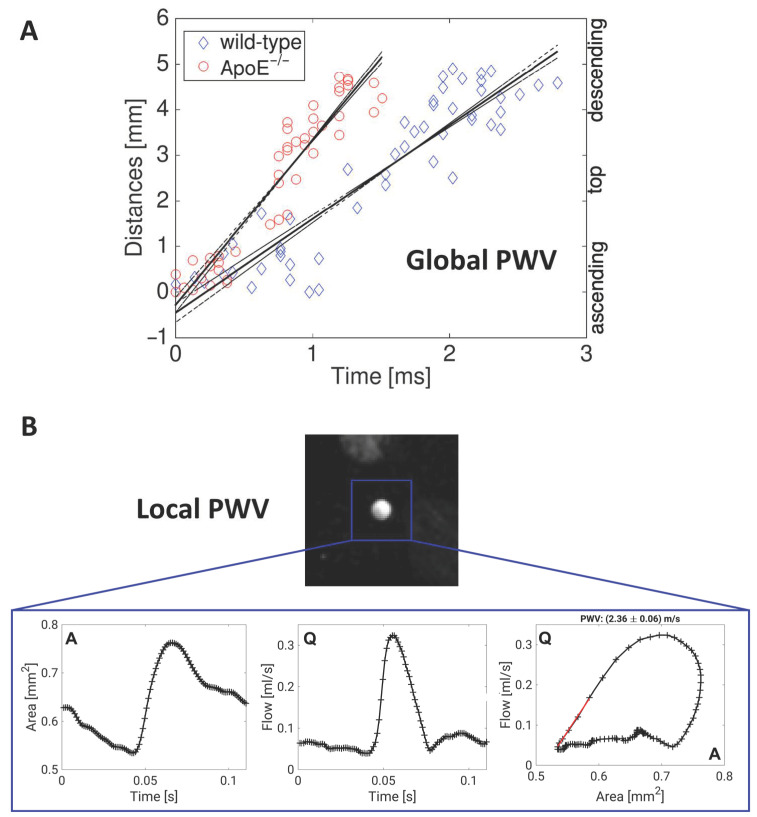
Global and local measurements of pulse wave velocity in murine vessels. (**A**) Assessment of global pulse wave velocity (PWV) for exemplary measurements in the aortic arch of a wild-type and an ApoE^−/−^ mouse. For determination of the global PWV values, the time points of the early systolic upstrokes were measured for multiple planes along the aortic arch (see Figure 1B) and plotted against the locations of the planes. The PWV is derived from the slope of a line fitted to the data points [80]. (**B**) Exemplary measurement of the local PWV in the abdominal aorta of a wild-type mouse using a retrospectively navigated PC-Cine MRI technique [121]. The through-plane flow (Q) and the cross-sectional areas (A) were determined. The local PWV is derived from the Q-A curve by fitting a line to the early systolic data points.

Due to the long measurement time and the required consistency of the heart cycle, the measurement of the local PWV is possible in only a few (2–3) locations. Therefore, a correlation of plaque-morphology with PWV is not possible in the whole aorta with conventional techniques. With a modified k-t BLAST (broad-use linear acquisition speed-up technique) method, the measurement time could be reduced to 1/8, allowing measurements of the PWV profile with the vessel morphology with a sampling density of 500 µm. With this technique, the measurement of the elasticity profile of the murine abdominal aorta could be realized for the first time, revealing a more heterogeneous distribution of PWV values in ApoE^−/−^ mice and pointing out the local character of lesion development [120]. Conventional cartesian imaging techniques are prone to disturbances due to motion and flow and, therefore, require synchronization with ECG- and respiratory probes. In contrast, radial k-space trajectories are more resistant to motion, as navigation signals of heart and breathing motion can be extracted from the radial k-space data, enabling an ECG-free retrospective reconstruction of dynamic image series (cines) and a more flexible data analysis, where also arrhythmias can be considered. This method was implemented on small animal MRI systems [121,122] and compared with conventional triggered measurement methods. By measuring the local PWV, the local vascular elasticity of the abdominal aorta could be assessed for the first time without additional heart or breathing signals (see Figure 2B) [121].

In the aortic arch, however, a complete profile is not obtainable due to the relatively high slice thickness and the curved geometry of the arch, especially in regions with high plaque susceptibility. Moreover, most of the flow velocity measurements are based on 2D-cine acquisition of single planes and the corresponding measurement of the velocity component perpendicular to it [68,89,119,120,121]. For a full determination of the flow dynamics in arteries, there is a need for 3D measurements with high temporal and spatial resolution, but in an adequate measurement time. In humans, 4D flow-sensitive MRI for the assessment of global aortic pulse wave velocity is already far advanced [123,124] and was validated in vitro [125]. Furthermore, it is already used in several clinical applications, for example in patients with aortic atherosclerosis [126]. Recently, it was also shown that the estimation of aoPWV with 4D flow MRI can be obtained and reproduced using a full 3D coverage in the human aortic arch. The results were compared and significantly associated with standard PWV acquisition [127]. Four-dimensional PC MRI was also used to determine PWV and wall shear stress in the aortic arch at the same time [128]. 

## 4. WSS and PWV Interact to Promote Plaque Growth and Vascular Inflammation

WSS and PWV are not separate factors with distinct functions; rather, there are mutual interactions that influence biological factors in the vessel wall. Various studies support the hypothesis that within the vascular microenvironment, a signaling cross talk occurs between arterial shear stress and vascular stiffness. Therefore, it is important to correlate altered hemodynamic properties like the PWV as a marker of vascular elasticity and the WSS locally. In a study using bovine aortic endothelial cells cultured on hydrogels, the cooperative effects of fluid shear stress and matrix stiffness were investigated. Hydrogels matching the elasticity of compliant (young) or stiff (aging) arteries were exposed to laminar fluid shear stress and revealed that endothelial cells grown on more compliant matrices displayed increased elongation and tighter endothelial cell junctions, decreased activation but enhanced nitric oxide production, suggesting that elastic matrices promote atheroprotective signaling [129]. Furthermore, disturbed flow not only drives endothelial cell activation, but it also causes arterial stiffening through factors released from endothelial cells, e.g., thrombospondin-1, which cause activation of profibrotic signaling pathways, leading to collagen deposition in murine and human arteries [130]. In addition, microcalcifications which are frequently detected within atherosclerotic lesions could bidirectionally affect WSS and PWV. By using a finite element analysis, it was found that even small microcalcifications can elevate the wall stress locally [19]. Interestingly, disruptions in the elastic fibers concomitantly observed with increased PWV [89] can feature a predisposition for the mineralization with calcium and phosphor [131]. 

### 4.1. Measurements of WSS, PWV, Plaque Characteristics and Inflammation

Four-dimensional PC MRI can overcome the limitations of conventional 2D-PWV measurements such as slice positioning in curved vessels like the aortic arch and through-plane susceptibility artifacts. One major bottleneck of 4D flow measurements, however, is the long acquisition time, which usually results in a compromise between spatial and temporal resolution. On the other hand, 4D PC-MRI provides a variety of advantages. For example, in certain vessels, global, regional, and local PWV can be assessed at the same time. Furthermore, other hemodynamic parameters such as wall shear stress, pressure gradients, oscillatory shear index and vessel geometry can be determined from the same measurement [132]. Therefore, 4D flow MRI and 3D PWV quantification is a promising tool for preclinical research, especially when used in mouse models of cardiovascular diseases. These murine disease models are used to advance our mechanistic understanding of atherosclerosis—thus, there is a need to adapt these methods to the murine system. In 2019, our group presented a self-gated radial 4D flow MRI sequence that enabled high-resolution WSS measurements in 35 min [79]. However, combined measurements of both WSS and PWV are challenging since both parameters have different requirements for the temporal and spatial resolutions [76]. In order to circumvent this issue, we introduced a new post-processing algorithm that allows the reconstruction of 4D flow cine datasets at variable temporal and spatial resolutions and the assessment of both PWV and WSS from the same MRI dataset (see Figure 1 and Figure 2A) [80]. This method was validated by in vivo measurements in wild-type and ApoE^−/−^ mice, showing good accordance with results for the PWV values in comparison to triggered methods [119]. 

However, for an even deeper insight into the processes causing atherogenesis, the heterogeneous distribution of plaques and elasticities needs to be considered [119]. Therefore, the ultimate goal of 4D PC-MRI is the acquisition of the local 3D PWV. The retrospectively gated radial 4D PC MRI sequence described above [80] may allow the assessment of local PWV values at certain locations of the aortic arch (proximal ascending aorta, distal descending aorta). The combined measurement of local PWV, global PWV, WSS and imaging of plaque characteristics and vessel wall inflammation may result in a better understanding of flow-related parameters with the loss of elasticity due to the continuous inflammation process during atherosclerosis progression. In the next chapter, a variety of different imaging techniques for visualization of plaque components and inflammation is described, which may be used in combination with the aforementioned flow-based MRI techniques in order to clarify the interrelation between hemodynamics, plaque composition and inflammation.

#### 4.1.1. Visualization and Differentiation of Structural Plaque Components with Contrast Weighting Techniques

**Figure 3:**
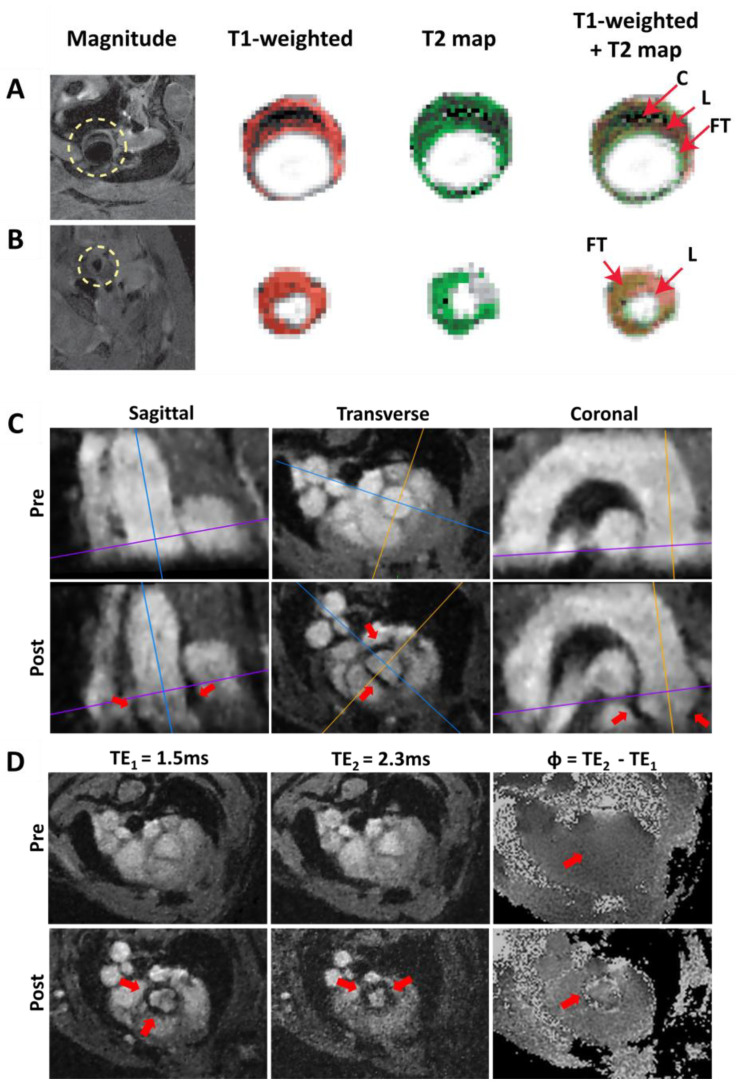
Morphology and inflammation imaging in murine atherosclerotic vessels. (**A**) Multispin multi echo (MSME) imaging of the ascending aorta (**A**) and the carotid artery (**B**) of an ApoE^−^/^−^ mouse fed a western diet. FG: Fibrotic tissue. L: Lipid rich core. C: Calcified tissue. (**C**,**D**) 3D VCAM-1 (vascular cell adhesion molecule-1) Measurement [141]. (**C**) Sagittal, transverse, and coronal orientation of the aorta extracted from the radial 3D-Cine measurement (TE_1_ = 1.5 ms) before administration of the contrast agent (pre) and after administration of the contrast agent (post). The lines mark the slice orientations. Areas of signal cancellation due to iron particles are marked with red arrows. (**D**) Measurement of the aortic arch at one exemplary position (see purple line in Figure 3C). Measurement with TE_1_ = 1.5 ms, TE_2_ = 2.3 ms and map of the phase differences Φ = TE_2_ − TE_1_. No significant phase jumps can be found in the aortic vessel wall in the pre-contrast agent measurement (red arrow, pre), whereas in the phase difference map of the post-contrast agent (CA) measurement (see red arrow in bottom right image (post)), significant phase jumps are observable.

Atherosclerotic plaques can contain different components, such as a lipid-rich necrotic core or intraplaque hemorrhage. The visualization and differentiation of these plaque components is possible with a variety of MR imaging techniques through the use of multiple contrast weightings, including T_1_-, T_2_-, T_2_*- or proton density- weighted contrast in ex vivo [133,134,135,136] and in vivo imaging applications [13,137,138,139], providing complementary information on the plaque composition and morphology. For example, lipid has hyperintense contrast in T_1_ weighted and proton density –weighted images and hypointense contrast in T_2_ weighted images, while calcified tissue has hypointense contrast in all acquisition techniques, as shown for an ApoE^−/−^ mouse fed a western diet in Figure 3A [140]. 

In ex vivo measurements of intracranial atherosclerotic plaques, Jiang et al. could classify different plaque types with an overall accuracy of 80.7% compared with histology through the assessment of the relaxation times (T_2_ and T_2_*) of the different plaque components [133]. In another ex vivo study using intracranial atherosclerotic plaque specimens, T_1_-, T_2_-, T_2_*- and proton density- weighted MRI at 7T was used in combination with histopathological validation for quantitative plaque characterization [136]. Here, significantly different relaxation times and proton density values were observed for different plaque components, with the best results for T_1_-weighted imaging for plaque characterization. Furthermore, intraplaque hemorrhages in coronary arteries of fixed human hearts were studied with T_1_- weighted MRI [134], while T_1_- and T_2_*- mapping was used to classify the components of surgically resected carotid plaques of patients [135]. MRI data were in good agreement with the corresponding histological sections. Quadratic discriminant analysis of the T_1_/T_2_* maps, however, resulted in a higher degree of misclassifications of plaques with inflammation or hemorrhage. 

For in vivo applications, fast T_1_- weighted spin echo techniques with compressed sensing were developed, enabling a faster and better visualization of the carotid artery wall and atherosclerotic plaques in patients [137]. Furthermore, T_2_ mapping was used to accurately quantify the lipid rich core in atherosclerotic carotid arteries, which was greater in symptomatic plaques than in asymptomatic plaques despite similar volume and luminal stenosis [138]. Moreover, carotid plaque lipid depletion after high-intensity statin treatment was successfully quantified in a following study [139]. For preclinical in vivo applications, atherosclerotic plaque stabilization was studied in rabbits using T_1_- and T_2_- weighted MRI in combination with ^18^F-FDG PET/CT [13].

#### 4.1.2. Gadolinium-Based Contrast Enhancement

Late gadolinium enhancement (LGE) and dynamic contrast enhancement (DCE) methods using gadolinium (Gd) based contrast agents (CA) are versatile tools to assess atherosclerotic plaque burden, to discriminate vulnerable plaques and to monitor vascular permeability. Gd contrast agents reduce T_1_ relaxation times and thus lead to a delayed signal enhancement of targeted areas in T_1_-weighted images. Late gadolinium enhancement using inversion recovery techniques was used to image vessel wall inflammation of Takayasu arteritis patients [142] and in atherosclerotic plaques [143]. Furthermore, Woodside et al. have visualized overall inflammatory cell burden in different regions of the aorta in ApoE^−/−^ mice at clinically relevant field strengths by using a targeted gadolinium contrast agent binding to the integrin α4β1, which is involved in the recruitment of inflammatory cells to atherosclerotic plaques [144]. Moreover, a recent study showed gadofluorine P accumulation in aortic atherosclerotic plaques of low density lipoprotein receptor deficient (Ldlr^−/−^) mice with MRI, followed by ex vivo mass spectrometry imaging to quantify the contrast agent to validate its local accumulation [145]. 

Another common Gd-based imaging technique is the DCE. Here, multiple images (before and after administration of the contrast agent), e.g., using black blood spin echo techniques are acquired. With this method, plaque microvasculature can be examined non-invasively in different human and animal studies, as reviewed in [146]. DCE was, furthermore, used for targeting macrophages in ApoE^−/−^ mice with gadolinium-containing synthetic lipopeptide nanoparticles [147], suggesting a high diagnostic value in the detection of vulnerable plaques. In another study, atherosclerotic plaque burden was assessed by T_1_ relaxivity measurements and volumetric analysis using gadospin F, showing significant correlations with histologlical 2D and 3D en face analyses of the aortas [148]. DCE-MRI is also capable of providing an early detection of high-risk plaques, as revealed in serial measurements in a rabbit model of atherothrombosis [149]. Here, disrupted plaques showed larger vessel wall areas and remodeling ratios as well as an increased gadolinium uptake in comparison to stable plaques. Another important application of DCE is the assessment of vascular permeability [150]. For instance, an albumin-binding contrast agent (gadofosveset) was used to monitor the permeability and remodeling after endothelial injury in wild-type and NOS3^−/−^ (endothelial nitric oxide synthase 3) mice [151] and to predict the risk of rupture in abdominal aortic aneurysms [152]. Furthermore, measurements using Gadomer-17 and Dotarem revealed elevated baseline permeability for Epac1^−/−^ (exchange protein directly activated by cyclic adenosine monophosphate (cAMP)) mice in contrast to the wild-type control group in a DCE-MRI study [153]. 

One downside of current Gd-based contrast agents is their toxicity [154]. Nevertheless, previous studies in humans revealed that Gd does not affect flow and PWV quantification [155] so that the combination of these techniques may be promising to gain a deeper understanding of the relationship between arterial hemodynamics and the corresponding inflammatory process, being responsible for plaque development and progression.

#### 4.1.3. Ultrasmall Superparamagnetic Iron Oxide Particle (USPIO)-Based Inflammation Imaging

Another approach to identify and visualize vascular inflammation sites during the progression of atherosclerosis is the use of ultrasmall superparamagnetic iron oxide particles (USPIOs) as a surrogate marker of macrophage accumulation and activity. These particles are engulfed by macrophages, showing their infiltration in the vascular wall through a rapid signal decline and, therefore, signal cancellations in T2* weighted images. To quantify the degree of the particle accumulation and, therefore, inflammation, pre- and post-contrast agent measurements need to be acquired and compared. Functionalized USPIOs against various targets were also successfully used in different human [156,157] and animal studies to visualize vascular inflammation. For example, imaging of ApoE^−/−^ mice with lipid-coated USPIOs targeting oxidation-specific epitopes or oxLDL-targeted iron oxide nanoparticles in the carotid arteries of ApoE^−/−^ mice demonstrated the successful imaging of atherosclerotic lesions [158,159]. Also, Scavenger receptor-AI–targeted iron oxide nanoparticles were able to detect atherosclerotic lesions in ApoE^−/−^ and Ldlr^−/−^ mouse models [160]. Furthermore, it is possible to image carotid plaque inflammation with Ferumoxytol, which is selectively taken up by atherosclerotic plaques [156]. In early atherosclerosis in particular, VCAM-1 is known to play a critical role. This adhesion molecule is overexpressed on the surface of inflamed ECs in atherosclerosis and mediates the recruitment and adhesion of immune cells to vascular inflammation sites. The use of functionalized USPIOs targeting VCAM-1, therefore, enables the visualization of these areas of arterial inflammation in atherosclerosis in animals [161] and in humans [157]. Moreover, dual-targeted microparticles of iron oxide (DT-MPIO) to detect VCAM-1 and P-selectin have furthermore been probed in mice [162]. Our group has demonstrated that USPIOs functionalized against VCAM-1 accumulated in the inflamed vessel wall, leading to a signal loss in T_2_* weighted images and enabling the visualization of early inflammation in the aortic arch [163]. 

Nevertheless, true imaging of vascular inflammation with MRI is still desirable since it would provide complete coverage of complex vessel geometries such as the aortic arch in contrast to 2D slices. Conventional methods to visualize these signal cancelations are based on triggered 2D FLASH measurements [163] and are usually limited to a few previously selected locations along the aorta, making a whole coverage of the vessel unfeasible. A further problem of 2D measurements is the usually large slice thickness, since objects with large susceptibility differences outside the image slice (e.g., lung tissue) can lead to misleading signal voids in the 2D image. 

As a proof of principle, we have further advanced the detection of USPIOs, based on an ECG-free radial multi-echo 3D-Cine measurement of the complete aortic arch (see Figure 3C) [141]. The cine reconstruction enables the tracking of the nanoparticles during the complete cardiac cycle and the measurement of two different gradient echoes provides the calculation of phase difference maps. These phase difference maps provide more detailed structure information when combined with magnitude maps to better assign the areas of VCAM accumulation. The post-CA measurement indicated significant signal losses in the aortic root and the ascending aorta due to the presence of the iron particles. In contrast, in the phase difference map from the baseline measurement, no significant local phase differences were detectable (see Figure 3D). This demonstrates that the coverage of the complete aortic arch at high spatial resolution and the detection of functionalized USPIOs is feasible with an ECG-free radial 3D-Cine acquisition [141]. In particular, when combined with mouse models of vulnerable plaques (e.g., cast models or the insulin-like growth factor-1 (IGF-1) knockout model, resulting in an unstable plaque phenotype in ApoE^−/−^ mice [164]), the 3D visualization of USPIOs to detect inflammation could provide the possibility to correlate the localization of inflammation sites with measurements of endothelial WSS and PWV in order to study its potential causal relationships and to characterize plaque vulnerability. 

## 5. Conclusions

Atherosclerosis is a chronic disease, initiated and modulated by blood flow characteristics, inflammatory processes in the vessel wall, and vessel wall elasticity. Low shear stress has been shown to promote arterial inflammation in multiple cell types to promote lesion formation and plaque vulnerability; the role of oscillatory shear stress seems more complex. Arterial wall stiffening is regulated by inflammatory processes and may in addition contribute to the inflammatory load. Moreover, WSS and PWV are intertwined in the regulation of biological processes in atherosclerosis. How these parameters correlate with disease and plaque vulnerability, however, remains to be defined. In this regard, MRI as a non-invasive technique—which does not use ionizing radiation—can be used to gather information on physical properties of flow and vessel wall properties. As we discussed here, cardiovascular MRI can already enable simultaneous measurements of flow-related parameters such as pulse wave velocity and wall shear stress. In the future, efforts should be made to further develop and improve the tools for the detection of inflammation. The analyses of these parameters in one measurement could significantly advance basic preclinical investigations of atherosclerotic lesion formation and progression, but also the diagnostic clinical evaluation of plaque characteristics to aid the early detection of culprit lesions which require therapy.

## Data Availability

The data presented in this study are available on request from the corresponding authors.
